# Unnecessary radiation exposure during diagnostic radiography in infants in a neonatal intensive care unit: a retrospective cohort study

**DOI:** 10.1007/s00431-022-04695-2

**Published:** 2022-11-10

**Authors:** Yu-Tsun Su, Yu-Shen Chen, Lee-Ren Yeh, Shu-Wen Chen, Yu-Cheng Tsai, Chien-Yi Wu, Yung-Ning Yang, Shu-Leei Tey, Chyi-Her Lin

**Affiliations:** 1 Department of Pediatrics, E-Da Hospital, #1, Yi-da Road, Jiaosu Village, Yanchao District, Kaohsiung, 82445 Taiwan; 2 School of Medicine for International Students, I-Shou University, Kaohsiung, Taiwan; 3 College of Medicine, I-Shou University, Kaohsiung, Taiwan; 4 Department of Radiology, E-Da Hospital, Kaohsiung, Taiwan; 5 Department of Medical Imaging and Radiological Sciences, College of Medicine, I-Shou University, Kaohsiung, Taiwan; 6 School of Medicine, I-Shou University, Kaohsiung, Taiwan; 7 Department of Nursing, E-Da Hospital, Kaohsiung, Taiwan; 8 Department of Pediatrics, National Cheng-Kung University Hospital, Tainan, Taiwan

**Keywords:** Babygram, Neonatal intensive care unit (NICU), Preterm, Radiation exposure, Radiography

## Abstract

Unnecessary radiation exposure (URE) during radiographic examination is an issue among infants in neonatal intensive care units (NICUs). The causes of URE have not been fully explored. This study investigated the incidence and identified the causes of URE in infants during diagnostic radiography in a NICU. This was a retrospective cohort study. We retrieved and analysed requests and radiographs taken at a tertiary NICU between September and November 2018. URE was defined as the rate of discordance between requests and images taken (DisBRI) and unnecessary radiation exposure in irrelevant regions (UREIR) during radiography. We compared the rates of URE between very low-birth-weight (VLBW, birth weight < 1500 g) infants and non-VLBW infants. A total of 306 radiographs from 88 infants were taken. The means ± standard deviations (SDs) of gestational age and birth weight were 35.7 ± 3.6 weeks and 2471 ± 816 g, respectively. Each infant underwent an average of 3.5 radiographs. The DisBRI rate was 1.3% and was mostly related to poor adherence to requests. The UREIR rates in thoraco-abdominal babygrams were 89.6% for the head, 14.8% for the elbows and 18.4% for the knee and were mainly related to improper positioning of and collimation in infants while performing radiography. The UREIR rates for the head, knee and ankle were higher in VLBW infants than in non-VLBW infants (94.6% vs. 85.6%, 27.0% vs. 11.5% and 5.4% vs. 0.7%, respectively, *p* < 0.05).

*Conclusions: *URE during diagnostic radiography is common in sick infants and is mainly related to improper positioning and collimation during examinations. Adherence to protocols when performing radiographic examination or using ultrasonography may be a solution to reduce URE in infants in NICUs.

**Table:** 

**What is Known:** *• The risk of unnecessary radiation exposure (URE) during radiography has been a common and important issue in sick infants in neonatal intensive care units (NICUs).* *• The new point-of-care ultrasound (POCUS) technique decreases the need for chest films and prevents radiation exposure in neonates.*	
**What is New:** *• In the NICU, URE is still a common issue in critically ill infants during radiographic examinations. The causes of URE during diagnostic radiography are mainly due to improper positioning and collimation during examinations.* *• The incidence of URE in irrelevant regions is higher in very low-birth-weight (VLBW) infants than in non-VLBW infants.*

**What is Known:**

*• The risk of unnecessary radiation exposure (URE) during radiography has been a common and important issue in sick infants in neonatal intensive care units (NICUs).*

*• The new point-of-care ultrasound (POCUS) technique decreases the need for chest films and prevents radiation exposure in neonates.*

**What is New:**

*• In the NICU, URE is still a common issue in critically ill infants during radiographic examinations. The causes of URE during diagnostic radiography are mainly due to improper positioning and collimation during examinations.*

*• The incidence of URE in irrelevant regions is higher in very low-birth-weight (VLBW) infants than in non-VLBW infants.*

## Introduction

Unnecessary radiation exposure (URE) in sick infants and children, particularly in very preterm infants, is a concern [1–3] because they may undergo multiple radiographic examinations in the neonatal intensive care unit (NICU) [4–6]. Furthermore, many irrelevant regions of the body are exposed to radiation during radiological examinations because of their small body size [2, 7]. The risk of radiation-induced damage to tissues is higher in infants and children than in adults at the same radiation dose [8–10]. Exposure to radiation in childhood has been shown to increase the risk of the development of solid cancers and leukaemia later in life [11, 12]. Many organisations have recommended the principle of As Low As Reasonably Achievable (ALARA) to reduce URE in infants during radiography [13, 14]. However, the effectiveness of this principle in reducing URE in very preterm infants is currently unclear.

Radiation-free point-of-care ultrasound (POCUS) has been used as an alternative diagnostic imaging technique in critical care [15–17]. Escourrou and De Luca first reported that the application of lung ultrasonography reduced the number of chest films and decreased radiation exposure in preterm infants in a NICU [18]. Despite evidence of the effectiveness of POCUS in critical care [19–21], radiography is still a common diagnostic tool in NICUs around the world [22–24].

This retrospective cohort study investigated the current incidence and analysed the causes of URE in infants who underwent diagnostic radiography in a NICU. We hypothesised that the smaller the infants, the more URE they had during hospitalisation.

## Materials and methods

### Study design, setting and participants

This was a retrospective cohort study. We enrolled infants who were admitted to the NICU of a university-affiliated hospital, which is a tertiary care centre. Radiography characteristics and data obtained from bedside diagnostic radiographic examinations performed between September and November 2018 were retrieved and analysed. Data including birth weight (BW), gestational age of the infant and total number of radiographs that each infant received in the NICU were collected. We used the Neonatal Therapeutic Intervention Scoring System (NTISS) to assess the severity of illness in all infants within 24 h of admission to the NICU [25]. The infants were divided into two groups: the very low-birth-weight (VLBW) group (those with a BW of < 1500 g) and the non-VLBW group (those with a BW of ≥ 1500 g). Two attending paediatricians assessed the images, reviewed the patients’ medical charts and identified URE, including the discordance between requests and images taken (DisBRI) rate and UREIR rate. If there was disagreement between the two paediatricians, a third paediatrician was consulted to reach a final conclusion. The incidence of URE was compared between the VLBW and non-VLBW groups. The study protocol was approved by the Hospital Ethical Review Committee (EMRP-107–137). This study followed the Strengthening the Reporting of Observational Studies in Epidemiology (STROBE) reporting guidelines [26].

### Inclusion criteria

Infants who were admitted to the NICU and underwent radiography during the study period were enrolled, and their medical records were retrieved and analysed. The types of radiographs included (1) thoraco-abdominal babygrams, defined as a radiograph with a large field of view, usually including the chest and abdomen; (2) radiographs specifically ordered to examine the location of peripherally inserted central catheters (RPICCs); (3) chest radiographs (anteroposterior [AP], left lateral and left decubitus); and (4) abdominal radiographs (AP and left decubitus).

### Exclusion criteria

Infants who did not undergo radiography were excluded.

### Protocol for bedside radiographic examinations

The protocol for radiographic examinations in the NICU was as follows: (a) A paediatrician ordered a bedside radiographic examination using the request form. (b) Nurses positioned the infant using a pillow and folded cloth towel. (c) A radiographer adjusted the machine parameters and collimation fields and obtained the radiograph using a mobile digital X-ray machine. (d) The image, which was usually electronically cropped, was stored on a picture archiving and communication system (PACS). (e) The paediatrician and radiologist read the image in the NICU and Department of Radiology, respectively.

All of the radiographic examinations were performed using a mobile digital X-ray System (Shimadzu Mobile Art Evolution X-ray machine; Shimadzu Corporation, Kyoto, Japan) with a total inherent filtration of 1.5 mm Al, focal spot size of 0.7 mm and tube target angle of 16°. A Fujifilm FCR imaging plate and cassette were used to obtain the radiographs, and an image reader (FCR PRIMA T2 (CR-IR 392), FujiFilm Corporation, Tokyo, Japan) was then used to process the film. For all radiographs, the tube voltage varied between 50 and 58 kVp, with a range of 1.6–2 mAs, and the source image receptor distance (SID) was set at 100 cm. The kVp, mAs and SID were set according to the instructions of the manufacturer.

### Recommended fields and craniocaudal and transverse collimation boundaries in radiographs

Paediatricians, radiologists and radiographers defined the regions of interest in the different types of radiographic examinations (Table 1) mainly based on the European Guidelines for Quality Criteria for Diagnostic Radiography in Paediatrics and the Radiopaedia website® [13, 27, 28]. The definitions were as follows: (1) The craniocaudal collimation boundaries and the fields of the radiographs were acceptable when thoraco-abdominal babygrams involved the chest, abdomen and pelvis and between the upper margin of the third cervical vertebra (C3) and 2 cm below the ischial tuberosities. (2) The transverse collimation boundaries and the fields of the radiographs were acceptable when the body regions observed were within 2 cm of the edge of the field. Tolerance for the maximal field size was defined as a distance of < 2 cm from the region of interest in children [13, 27–29]. We did not define the recommended field in RPICCs because the appropriate regions of radiation exposure varied widely from the line insertion site to the tip of the long line.

**Table 1:** Recommended fields for different types of radiographs, mainly based on the European Guidelines for Quality Criteria for Diagnostic Radiography in Paediatrics

Type of radiograph	Region of interest	Craniocaudal boundaries		Transverse boundaries
		Upper border	Lower border	Right and left border
Thoraco-abdominal babygram	Lung, abdomen, pelvis	C3	< 2 cm below the ischial tuberosities	< 2 cm from the side of trunk
AP chest	Lung	C3	T12/L1	< 2 cm from the side of trunk
AP abdomen	Abdomen, pelvis	< 2 cm above the diaphragm	< 2 cm below the ischial tuberosities	< 2 cm from the side of trunk

*AP *anterior–posterior, *C3 *the third cervical vertebra, *T12 *the 12th thoracic vertebra, *L1 *the first lumbar vertebra

### Number of radiographs per infant

The total number of radiographs that each infant received in the NICU during the study period was collected.

### Rate of discordance between requests and images taken

The rate of DisBRI has been used to monitor discordance between requests and images taken and has been related to poor adherence to the procedure protocol. [30–32]. A discordant radiograph was defined as a specific radiograph that as discordant with the paediatrician’s request. For example, a paediatrician ordered a single chest radiograph, but a nurse or radiographer performed a thoraco-abdominal babygram. The rate of DisBRI was defined as the number of discordant radiographs divided by the total number of radiographs performed.

### Rates of unnecessary radiation exposure of irrelevant regions and upper and lower borders beyond the recommended collimation fields

The body parts that were expected to be included in the radiograph were defined as regions of interest. When a body region other than the region of interest was observed in the radiograph, it was considered UREIR. The rate of UREIR has been related to improper positioning of and collimation in infants while taking radiographs [33–35]. UREIR to the elbow, wrist, knee and ankle was defined as total exposure in the distal part of the proximal bone of the joint or partial exposure in the proximal part of the distal bone during radiography. The rate of UREIR for each region was defined by dividing the number of radiographs in which that region was observed by the number of radiographs of each type according to paediatricians’ orders. The rates of the upper, lower, right and left borders of the images exceeding the recommended range were also calculated. We did not analyse the UREIR in infants with RPICCs.

### Statistical analysis

Statistical analysis was performed using SPSS version 17.0 (SPSS Inc., Chicago, IL, USA). Categorical variables are presented as frequencies and percentages. Data distribution was evaluated by the Kolmogorov‒Smirnov test and Shapiro‒Wilk test. Numerical variables that followed normality assumptions were analysed using Student’s *t* test, and those that violated normality assumptions were assessed using the Mann‒Whitney *U* test. We compared the rates of DisBRI and UREIR between the VLBW and non-VLBW groups using the chi-square test. We used Fisher’s exact test when subject counts were less than 5. A *p* value of < 0.05 was considered to be statistically significant.

## Results

### Characteristics of the infants and radiographs

**Fig. 1:**
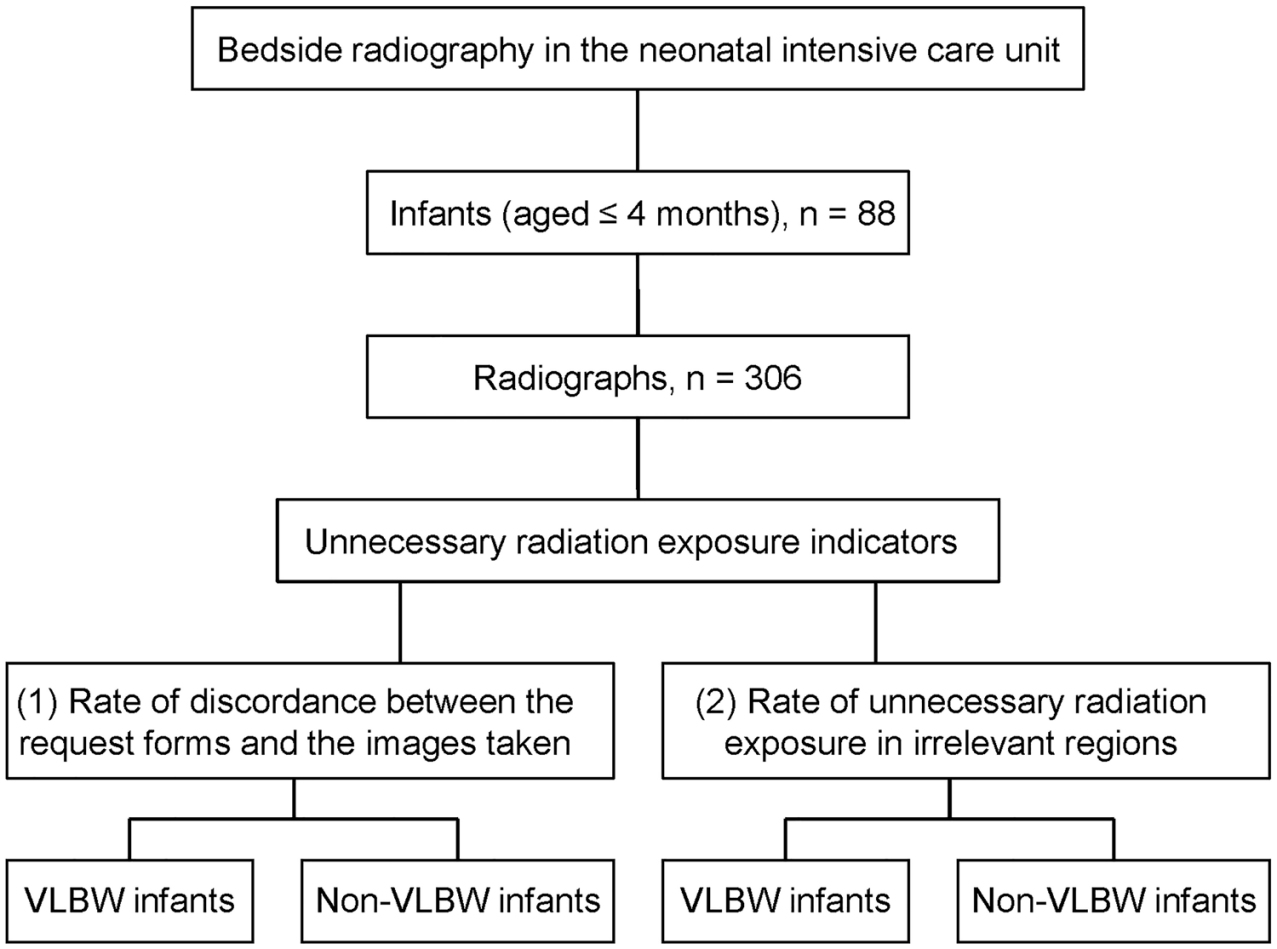
Study flowchart. (VLBW, very low-birth-weight, birth weight < 1500 g)

A total of 88 infants were enrolled, and 306 images were retrieved (Fig. 1). The characteristics of the overall cohort and the VLBW and non-VLBW groups are shown in Table 2. The infants’ mean ± standard deviation (SD) birth weight was 2471 ± 816 g, and the mean ± SD gestational age was 35.7 ± 3.6 weeks. The mean NTISS score was higher in the VLBW group than in the non-VLBW group.

**Table 2:** Clinical characteristics of the participants in the overall cohort and the VLBW and non-VLBW groups

Characteristic	Overall	VLBW	Non-VLBW	*p* value
**Case number, *n***	88	12	76	
**Sex**				
Male, *n* (%)	48 (54.5)	6 (50.0)	42 (55.3)	0.764
Gestational age, weeks*				
Mean ± SD	35.7 ± 3.6	29.2 ± 3.0	36.8 ± 2.3	< 0.001*
Median (IQR 25–75%)	36.8 (33.6–38.2)	29.0 (26.4–32.3)	37.1 (35.4–38.4)	
Birth weight, g*				
Mean ± SD	2471 ± 816	1122 ± 308	2684 ± 650	< 0.001*
Median (IQR 25–75%)	2593 (1848–3096)	1080 (835–1453)	2758 (2159–3146)	
**Apgar score at 5 min**^**#**^				
Mean ± SD	8.4 ± 1.0	7.3 ± 0.9	8.6 ± 0.9	
Median (IQR 25–75%)	9 (8–9)	7.5 (7–8)	9 (8–9)	< 0.001^**#**^
**NTISS**^**#**^				
Mean ± SD	14.7 ± 5.3	19.3 ± 4.8	14.0 ± 5.0	
Median (IQR 25–75%)	13 (12–15)	19.5 (14.3–23.5)	13 (12–14)	< 0.001^**#**^
**Average number of radiographs per infant**^**#**^				
Mean ± SD	3.5 ± 6.9	11.4 ± 15.5	2.2 ± 2.7	
Median (IQR 25–75%)	1 (1–3)	4 (3–10.5)	1 (1–2)	< 0.001^**#**^

The *p* value was calculated for the VLBW group vs. the non-VLBW group, A *p* value of < 0.05 was considered to be statistically significant

*VLBW *very-low-birth weight, *NTISS *Neonatal Therapeutic Intervention Scoring System

*Followed normality assumptions and were analysed using Student’s *t* test

^#^Violated normality assumptions and were assessed using the Mann‒Whitney *U* test

### Number of radiographs obtained per infant in the overall cohort and the VLBW and non-VLBW groups

Each infant received an average of 3.5 ± 6.9 radiographs (median: 1, interquartile range [IQR] 25–75%: 1–3); 11.4 ± 15.5 (median: 4, IQR 25–75%: 3–10.5) in the VLBW group and 2.2 ± 2.7 (median: 1, IQR 25–75%: 1–2) in the non-VLBW group (Table 2). The VLBW group underwent significantly more radiographic examinations than the non-VLBW group (*p* < 0.001).

### Rates of DisBRI in the overall cohort and the VLBW and non-VLBW groups

The numbers of the different types of radiographs categorised by requests of paediatricians and by the radiographs obtained are shown in Table 3. When the types of images obtained were compared to the requests, the overall rate of DisBRI was 1.3%. It was higher in the VLBW group (2.2%) than in the non-VLBW group (0.6%); however, this difference was not significant (*p* = 0.22). We found four chest AP radiographic orders were mis-performed as babygrams. We then interviewed the corresponding medical staff about the cause of DisBRI and disclosed that it was due to poor adherence to the radiographic protocol.

**Table 3:** Number of different radiographs according to paediatricians’ requests and obtained images and the rate of discordance between requests and images taken (DisBRI) in the overall cohort and the VLBW and non-VLBW groups

	Paediatricians’ requests (*n* = 306)			Obtained images (*n* = 306)		
	Overall	VLBW	Non-VLBW	Overall	VLBW	Non-VLBW
Babygram	250	111	139	254	114	140
RPICC	45	18	27	45	18	27
Chest AP	5^a^	4^e^	1^ h^	1^b^	1^d^	0^ g^
Chest (left decubitus)	1	1	0	1	1	0
Chest (left lateral)	1	0	1	1	0	1
Abdomen AP	0	0	0	0	0	0
Abdomen (left decubitus)	4	3	1	4	3	1
Total (*n*)	306^c^	137^f^	169^i^	306	137	169
	Overall		VLBW	Non-VLBW	*p* value^f^	
DisBRI rate (%)^k^	1.3		2.2	0.6	0.22	

*VLBW *very low-birth-weight, *RPICC *radiographs specifically ordered to check the location of peripherally inserted central catheters, *AP *anterior–posterior

^j^The *p* value was calculated for VLBW infants versus non-VLBW infants

^k^The DisBRI rate was calculated as (*a* − *b*)/*c* = (5 − 1)/306 = 1.3% in the overall cohort, (*e* − *d*)/*f* = (4 − 1)/137 = 2.2% in the VLBW group and (*h* − *g*)/*i* = (1 − 0)/169 = 0.6% in the non-VLBW group (*p* = 0.22)

### Rates of UREIR and upper and lower borders beyond the recommended collimation fields in the overall cohort and the VLBW and non-VLBW group

**Fig. 2:**
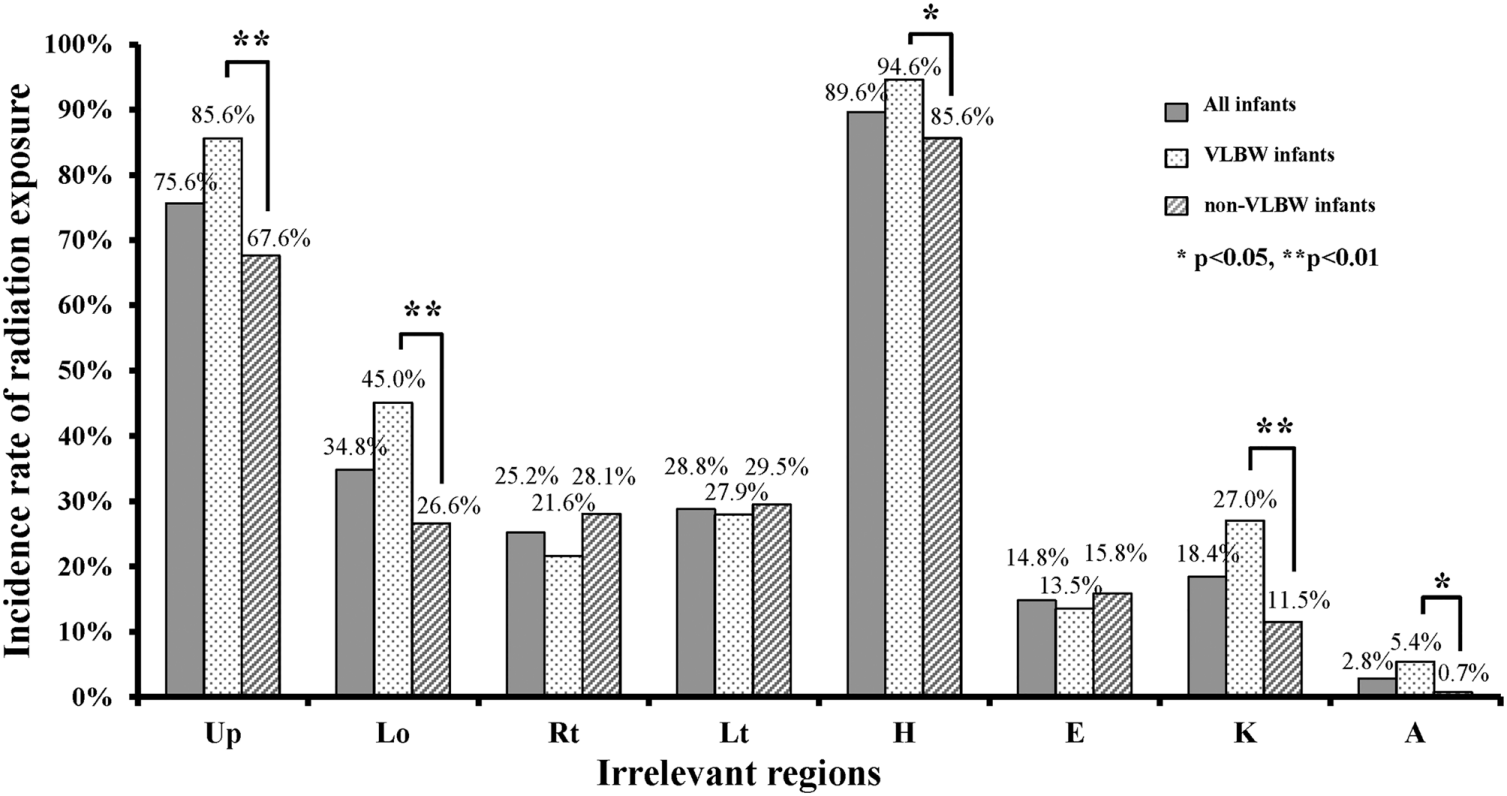
The rate of unnecessary radiation exposure in irrelevant regions and the rates of the upper, lower, right and left borders of the images exceeding the recommended fields in thoraco-abdominal babygrams. (Up, upper; Lo, lower; Rt, right; Lt, left; H, head; E, elbow; K, knee; A, ankle)

UREIR was assessed in 250 babygrams according to paediatricians’ orders. The UREIR rates and the rates of images beyond the craniocaudal and transverse borders of the recommended collimation fields are shown in Fig. 2. The UREIR rate was 89.6% for the head in the overall cohort. The UREIR rates for the head, knee and ankle in the VLBW group were significantly higher than those in the non-VLBW group (Fig. 2). The rates of upper and lower borders beyond the recommended collimation field were also significantly higher in the VLBW group than in the non-VLBW group (Fig. 2). Due to few cases, we did not analyse the UREIR rates in infants who underwent chest radiographs taken in the anterior–posterior (AP) and lateral (*n* = 7) positions and abdominal radiographs taken in the AP and decubitus (*n* = 4) positions.

## Discussion

This retrospective cohort study investigated the incidence and analysed the causes of URE during diagnostic radiography in critically ill infants. We found that critically ill infants still undergo frequent radiographic examinations in a NICU. URE was common and mainly due to improper positioning and collimation during examinations, particularly in VLBW infants.

Preterm infants are susceptible to morbidities such as respiratory distress syndrome, bronchopulmonary dysplasia, necrotising enterocolitis, sepsis and patent ductus arteriosus and require radiographic examinations for diagnosis and management during their hospital stay [4–6, 30]. The improvement in the survival rate of preterm infants has raised concerns regarding URE in NICUs. The European Commission, American College of Radiology (ACR), Society for Pediatric Radiology (SPR) and Society of Thoracic Radiology (STR) have adopted the ALARA principle and implemented recommendations to reduce URE [13, 14]. However, these guidelines are not specifically designed to address bedside radiography in infants in NICUs. Therefore, this study analysed quality indicators of radiography, including (1) the number of radiographs taken per infant, (2) the rate of DisBRI and (3) the rate of UREIR and investigated the causes of URE in critically ill infants.

We found that each infant had an average of 3.5 radiographs taken during a 3-month period in the NICU. The mean number of radiographs per infant in our study was similar to those reported in other studies [2, 30]. Although the optimal number of radiographs varies by infant and depends on the underlying disease and its severity, a severely ill infant generally receives multiple radiographic examinations with a consequently accumulative radiation dose. In a previous study, after a NICU radiation safety programme was implemented, the mean number of radiographs per patient was reduced from 4.2 to 1.9 radiographs within 5 years [32]. Thomas et al. found that only 14% of paediatricians could recall training on the hazards of medical radiation [36]. Ekşioğlu et al. also reported that 89% of paediatricians were not aware of the ALARA policy [31]. Hence, educating front-line paediatricians about the indications and guidelines for radiographic examinations is crucial.

Escourrou and De Luca used lung ultrasonography as the first-line imaging technique and chest radiography as the second-line examination in infants with respiratory distress in the NICU and successfully reduced the mean number of chest radiography per infant from 4.9 to 2.6. In addition, the mean radiation dose per infant was reduced from 183 to 68 μGy, and the percentage of infants who underwent chest radiography was reduced by 11% (from 81 to 69.7%) [18, 37]. In 2020, the European Society of Paediatric and Neonatal Intensive Care (ESPNIC) issued evidence-based POCUS guidelines to optimise POCUS use in diagnosis and procedural performance in critically ill neonates and children [16]. Nowadays, the POCUS technique has been integrated into clinical protocols and suggested as a first-line tool to replace conventional radiography in infants in many NICUs [18, 37].

The overall DisBRI rate was 1.3% in this study. Four chest AP radiographs were ordered, but the radiographers performed babygrams instead. We found that it was related to poor adherence to medical orders, partially due to insufficient communication between paediatricians and staff about the clinical conditions of patients and the habits of radiographers who were used to taking thoraco-abdominal babygrams regardless of the type of order [30–32]. Based on the potential radiation hazard, AP chest and AP abdominal radiographs should be taken according to respective indications and requests [14]. Edison et al. reported a DisBRI rate of 18% in a neonatal unit in 2015; and the rate decreased to 4% 6 months after implementation of a radiation safety project [30].

Bader et al. found that the UREIR rates in thoraco-abdominal babygrams were 37% for the head, 62% for the thigh, 26% for the knee and 1% for the ankle [2]. Pederson et al. reviewed 100 neonatal chest radiographs and found that 30% were above the upper border and 20% were below the lower border of the recommended fields [29]. Evidenced by these results, UREIR is still common and is mostly related to improper positioning of and collimation in infants. Nursing staff usually use restraint devices to prevent patients from moving during examinations, thus minimising UREIR [34, 35, 38]. Currently, there are no standardised anatomical landmarks for radiographers to use as a reference. Therefore, they usually widen the collimation fields to include all body regions of interest in radiographic examinations [13, 29, 39, 40]. Graham and Hardy reported that 73.3% of radiographers need guidelines for restraining patients and that 84.6% of them require further training [33]. In addition, Datz et al. reported that at least a 50% reduction in UREIR can be achieved if a radiograph is obtained by using a proper collimation technique [41]. The optimal position and use of restraint devices for infants and using precise surface landmarks for collimation are crucial to reduce UREIR [7, 42].

VLBW infants were sicker and had higher NTISS scores than non-VLBW infants and received 5 times more radiographs than the latter in this study. The rates of UREIR for the head, knee and ankle and the rates of upper and lower borders beyond the recommended collimation fields were also significantly higher in VLBW infants. These findings have not been reported previously [43, 44].

The “European guidelines on quality criteria for diagnostic radiographic images in paediatrics” and “ACR-SPR-STR practice parameter for the performance of portable chest radiography” address radiographic dose/technique settings, image clearness and contrast, and collimation fields in conventional radiography [13, 14]. However, these guidelines mostly target radiologists and medical physicists rather than paediatricians, nursing staff and radiographers. Potential guidelines for image study in NICUs should include (1) qualified medical professionals trained on the ALARA concept; (2) application of POCUS first; (3) request the correct type of radiograph according to the clinical indication by paediatricians; (4) effective communication to staff about the order; (5) performance of the correct radiograph according to the requests; (6) the use of appropriate devices to help position and restrain the patient; (7) establishment of recommended technique/dose settings and surface anatomical landmarks for collimation; and (8) periodic monitoring of quality indicators for radiographic examinations.

URE occurs during daily practice in critically ill infants. Similar errors in neonatal care, such as infusion pump programming, mis-calculation of surfactant dose and medication errors, occur in NICU [45–47]. Continuously monitoring the incidence of errors in healthcare, exploring the underlying mechanisms and implementing prevention strategies are needed to assure healthcare quality in neonatal care [32, 45, 47].

Although this was a retrospective cohort study, it has several strengths. First, we used various quality indicators to monitor URE associated with bedside diagnostic radiography in infants in the NICU, particularly in VLBW infants. Although the results were collected at only one medical centre, the results of URE deserve attention. Second, we investigated the whole process of performing radiography, including prescribing the order, performing the radiographic examination and analysing the roles of different medical professionals involved in the process, which has not been done before. However, there are limitations of this study. First, we analysed radiographs stored in the PACS, which were usually electronically cropped from the initial portable image; therefore, the actual UREIR rate is expected to be higher than that reported in the present study. Second, data on the number of repeated radiographs due to a failed first examination were not available in the PACS and therefore were not included in the analysis. Third, the accumulated radiation dose (μGy) in infants was not collected for analysis. Fourth, we did not analyse patients’ detailed morbidities and the severity of their condition. Therefore, a further study to assess original images before electronic cropping along with radiation dose and detailed patient information is needed to continuously evaluate URE from conventional radiography in critically ill infants.

In conclusion, URE was common in infants cared for in the NICU. Although the percentage of DisBRI was low, a high rate of UREIR occurred in infants undergoing radiography, particularly VLBW infants. Improper positioning and restraint of infants, as well as improper collimation during examinations, were major contributing factors. Implementing a protocol including POCUS as the first-line imaging tool of choice before performing a radiographic examination may reduce URE. Training medical staff to adhere to guidelines and follow standard protocols for diagnostic radiography is urgently needed.

## Data Availability

The patients’ radiographs and clinical information used to support the findings of this study are stored in the PACS and Medical Information System in E-Da hospital. According to the regulation of E-Da Hospital Ethical Review Committee and the “Personal Information Protection Act” in Taiwan, patients’ raw data cannot be made publicly available. The interpretation of radiographs and clinical information that has been unlinked to patients is available from the corresponding author upon request of the editorial staff.

## Abbreviations

ACR: American College of Radiology
ALARA: As Low As Reasonably Achievable
AP: Anteroposterior
BW: Birth weight
DisBRI: Discordance between requests and images taken
IQR: Interquartile range
NICU: Neonatal intensive care unit
NTISS: Neonatal Therapeutic Intervention Scoring System
PACS: Picture archiving and communication system
RPICC: Radiograph for peripherally inserted central catheters
SID: Source image receptor distance
SPR: Society for Pediatric Radiology
STR: Society of Thoracic Radiology
URE: Unnecessary radiation exposure
UREIR: Unnecessary radiation exposure in irrelevant region
VLBW: Very low-birth-weight
